# The effectiveness of a clinically integrated e-learning course in evidence-based medicine: A cluster randomised controlled trial

**DOI:** 10.1186/1472-6920-9-21

**Published:** 2009-05-12

**Authors:** Regina Kulier, Sjors FPJ Coppus, Javier Zamora, Julie Hadley, Sadia Malick, Kausik Das, Susanne Weinbrenner, Berrit Meyerrose, Tamas Decsi, Andrea R Horvath, Eva Nagy, Jose I Emparanza, Theodoros N Arvanitis, Amanda Burls, Juan B Cabello, Marcin Kaczor, Gianni Zanrei, Karen Pierer, Katarzyna Stawiarz, Regina Kunz, Ben WJ Mol, Khalid S Khan

**Affiliations:** 1 The University of Birmingham, Edgbaston, Birmingham B15 2TG, UK; 2 Academic Medical Center, University of Amsterdam, Department of Obstetrics and Gynaecology, Meibergdreef 9, 1105 AZ Amsterdam, the Netherlands; 3 Academic Medical Center, University of Amsterdam, Department of Clinical Epidemiology and Biostatistics, Meibergdreef 9, 1105 AZ Amsterdam, Amsterdam, the Netherlands; 4 Clinical Biostatistics Unit, Hospital Ramon y Cajal, Ctra Colmenar, km 9.100 28034, Madrid, Spain; 5 Birmingham Women's Hospital, Metchley Park Road, Edgbaston, Birmingham, B15 2TG, UK; 6 Heart of England NHS Foundation Trust, Solihull Hospital, Lode Lane, Solihull, B91 2JL, UK; 7 Agency for Quality in Medicine, Weglelystrasse 3, 10623 Berlin, Germany; 8 University of Pécs, Department of Paediatrics, József Attila u. 7, Pécs, H-7623, Hungary; 9 TUDOR, University of Szeged, Albert Szent-Gyorgyi Medical and Pharmacological Centre, Somogyi Bela ter 1, Szeged, H-6725, Hungary; 10 CASPe (CASP Espana), Joaquin Orozco 6, 1°-F, 03006 Alicante, Spain; 11 CASPolska, 30–347 Krakow, ul. Wadowicka 3, Poland; 12 Universitá Cattolica del Sacro Cuore, Via Emilia Parmense 84, 29100 Piacenza, Italy; 13 Basel Institute for Clinical Epidemiology, Hebelstrasse 10, CH 4031 Basel, Switzerland; 14 CIBER Epidemiologia y Salud Publica (CIBERESP), Barcelona, Spain

## Abstract

**Background:**

To evaluate the educational effects of a clinically integrated e-learning course for teaching basic evidence-based medicine (EBM) among postgraduates compared to a traditional lecture-based course of equivalent content.

**Methods:**

We conducted a cluster randomised controlled trial in the Netherlands and the UK involving postgraduate trainees in six obstetrics and gynaecology departments. Outcomes (knowledge gain and change in attitude towards EBM) were compared between the clinically integrated e-learning course (intervention) and the traditional lecture based course (control). We measured change from pre- to post-intervention scores using a validated questionnaire assessing knowledge (primary outcome) and attitudes (secondary outcome).

**Results:**

There were six clusters involving teaching of 61 postgraduate trainees (28 in the intervention and 33 in the control group). The intervention group achieved slightly higher scores for knowledge gain compared to the control, but these results were not statistically significant (difference in knowledge gain: 3.5 points, 95% CI -2.7 to 9.8, p = 0.27). The attitudinal changes were similar for both groups.

**Conclusion:**

A clinically integrated e-learning course was at least as effective as a traditional lecture based course and was well accepted. Being less costly than traditional teaching and allowing for more independent learning through materials that can be easily updated, there is a place for incorporating e-learning into postgraduate EBM curricula that offer on-the-job training for just-in-time learning.

**Trial registration:**

Trial registration number: ACTRN12609000022268.

## Background

Evidence-based medicine (EBM) requires healthcare professionals to engage with contemporaneous research evidence in clinical decision making.[1] To achieve this, EBM curricula need to inculcate amongst learners the skills to acquire, assess and apply new knowledge in the clinical setting. However, training programs to improve evidence-based decision making have generally not been robustly evaluated. There has been much debate about the various EBM teaching and learning methods, but now there is consensus that best educational practice should be clinically integrated.[2] It should result not only in improvements in knowledge and appraisal skills, but also in attitudes and behaviour, which in the end leads to improved practice.[2]

Various models of teaching and learning strategies have been described, taking into account the different needs of adult learners, such as self-directed and problem-centred learning. [3]

However, traditionally, postgraduate education is generally delivered via lecture-based courses and workshops which do not directly exploit learning opportunities in the workplace. The EU-EBM Unity project was developed to address these shortcomings. [4] The project was conducted within the European Union's Leonardo da Vinci vocational training programme involving 11 European partners. [4,5] We used established methodology to develop a learner-centred, problem-based course that employs e-learning to provide on-the-job training exploiting learning opportunities during the course of clinical activity. [4-6] In brief, the course consists of five modules, to be followed consecutively and is outlined in table 1. Each module consists of a self-directed e-learning part and practical assignments that have to be completed and discussed with the facilitator before moving on to the next module. Our piloting showed that this course can be adapted to different clinical settings in different languages and it is well accepted by the trainees and tutors. [5]. In this trial, we set out to evaluate the educational effectiveness of our clinically integrated e-learning course for teaching basic EBM to postgraduates compared to a traditional lecture based course of equivalent content in terms of knowledge gain and change in attitude towards EBM.

**Table 1:** Overview of the clinically integrated e-learning course compared to lecture based course in evidence-based medicine (EBM)

**Clinically integrated e-learning course and Lecture based course**	

**Aim: **To familiarise course participants with evidence based medicine (EBM) basics	

**Target participants: **Health professionals in a clinical setting.	

**Learning objectives:**	
Upon the completion of the course, participants should be competently able to:	
generate structured questions arising from clinical problems in practice	
search relevant literature, identifying systematic reviews wherever possible	
assess the quality (validity) of systematic reviews and primary research included within them	
assess the applicability of research findings in clinical practice	
effectively implement the output from above activities into clinical practice	

**E-learning modules:**	
Five models provide learning materials at	
Module 1: Asking clinical questions	
Module 2: Searching the evidence	
Module 3: Critical appraisal of systematic reviews (and their constituent studies)	
Module 4: Applicability of the evidence to the patient	
Module 5: Implementation of evidence into practice	

**Assessment:**	
Multiple choice questions to test knowledge and questionnaire to test attitudes	

**Clinically integrated e-learning course**	**Lecture based course**

**Learning/teaching methods**	

Knowledge needs identification in the clinical setting	Traditional lecture-based course using the power point slides from the e-learning modules
Participants to pursue independent study by using the e-learning modules	Lectures presented during 2–4 sessions
Interaction with facilitator throughout the course	Interaction with the tutor only during lecture

## Methods

We conducted a cluster randomised controlled trial [7] to compare the clinically integrated e-learning EBM course to a lecture-based course for its effect on knowledge and attitudes after obtaining approval from the relevant authorities. We chose this approach to avoid the risk of contamination, i.e. the inadvertent delivery of intervention to members of the control group, associated with individual randomisation in educational research.[8] Random allocation sequence was generated by computer to either intervention or control group and was stratified by country. The trial procedures were tested for feasibility in Switzerland in different clinical departments, after which the study was conducted in four centres in the Netherlands and two centres in the United Kingdom between August and December 2007. The participants were obstetrics and gynaecology trainees in clinical teaching hospitals who did not rotate between clusters during the study period. The trainees were all junior medical doctors who had not previously received formal EBM teaching in their postgraduate training. All trainees provided consent for use of their data anonymously at the start of the trial.

The clinically integrated course consisted of five modules, each comprising self-directed e-learning components and clinically related activities, under the guidance of a facilitator (table 1). The curriculum , described in detail elsewhere,[4] was delivered at each site over a 4–6 week time period. In the control group, the material covered in the e-learning module was presented by a tutor during classical lecture-based teaching sessions over the same time period. The tutors presented from the same power point slides used in the intervention group. We assessed gain in knowledge and change in attitude towards EBM by comparing pre and post intervention scores. We adapted previously validated questionnaires for measurements of knowledge and attitudes which is described in detail elsewhere. [4,9-11] We adapted these questions to match the content and learning objectives of our course. The maximum possible knowledge score was 62 points. Responses to questions about attitude towards EBM were possible on a five point Likert-scale, ranging from 'strongly agree' to 'strongly disagree'.

Data were collected online for the intervention group and on paper for the control group. Before starting the course, trainees in both groups completed all knowledge and attitude questionnaires. Post-course questions were completed immediately after each lecture in the control group. In the intervention group, trainees could complete the questions in their own time after completing the various modules of the course. Additional materials were not allowed to be used during the exams. Attitude questionnaires were re-administered at the end of the course in both groups.

Responses to the knowledge questionnaires were scored and between group comparisons were made taking into account the cluster design of the study. Characteristics of individual doctors in the same cluster, as well as their scores, are likely to be correlated. This correlation should be taken into account in the analyses. Generalized estimating equations (GEE)[9] allow to extend linear models to take into account the correlation between individuals in the same cluster. For a cluster randomized trial, this matrix is assumed to be exchangeable, i.e. all correlations between individuals in the same cluster are the same. The proposed model could also be extended to adjust for individual covariates at baseline. Thus, in our model, the dependent variable was the final score and the intervention and baseline scoring were the independent variables. Attitudinal gain was defined as any change of whatever magnitude towards a more positive attitude towards EBM as measured with a Likert scale. All analyses were performed using Stata (StataCorp. 2007. Stata Statistical Software: Release 10. College Station, TX: StataCorp LP).

## Results

**Figure 1:**
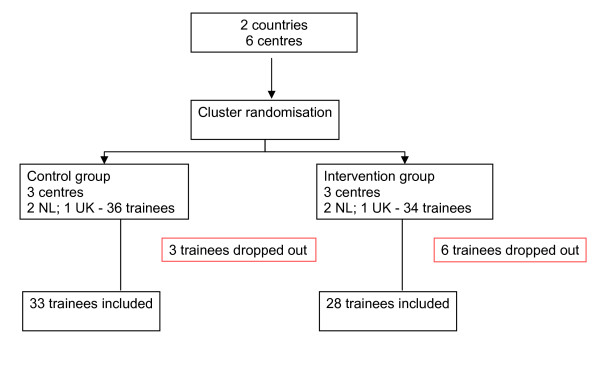
**Flow chart of participants in the trial**.

**Figure 2:**
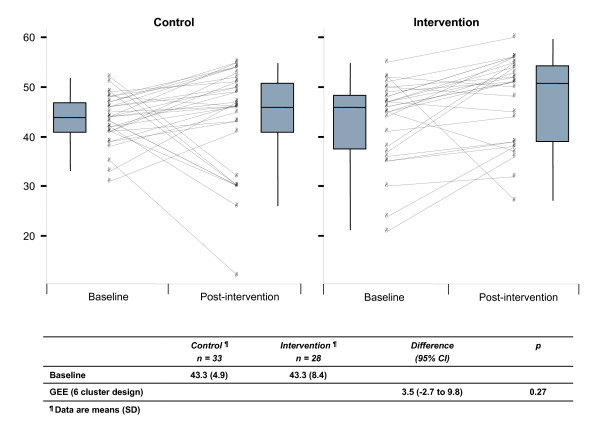
**Pre-and post-course scores adjusted for baseline knowledge**.

**Figure 3:**
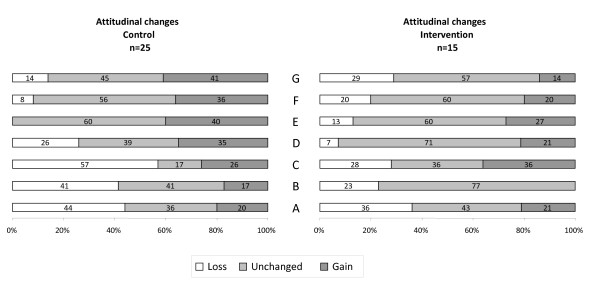
**Attitudinal gain**. (A): Original research is confusing (B) Study design is important in article selection (C) Evidence-based decision making is ' health care by numbers' (D) Contracts for health care professionals should include time taken away from patient care for reading and appraising the literature (E) I am confident that I can assess research evidence (F) Systematic reviews play a key role in informing evidence-based decision making.

The total number of postgraduate trainees included in the analysis was 61, 28 in the intervention group and 33 in the control group (figure 1) recruited from six clusters (three clusters in each arm). The baseline knowledge scores were the same for the intervention and control groups and were generally high (43.3 points corresponding to 70% of the maximum possible scoring). Post-course scores were improved in both groups compared to baseline (figure 2). After adjusting for baseline knowledge, the intervention group outperformed the control group by 3.5 scoring points (95% CI -2.7 to 9.8) but this difference was not statistically significant (figure 2). In relative terms the difference was 5.6% (95% CI -4.4 to 15.8). About 18% of trainees in the intervention group and 27% in the control group had lower scores post course than pre course. Although a slightly higher proportion of participants in the control group showed an attitudinal gain towards EBM compared to the intervention group, the low number of participants did not allow to show any statistically significant difference (figure 3).

## Discussion

The main findings of our study showed that both teaching approaches lead to improvement in basic EBM knowledge. The clinically integrated e-learning course produced slightly better scores compared to the classical teaching approach; however, this difference did not reach statistical significance. Attitudinal gains did not differ between the groups. Although further evidence is needed, one could hypothesize that equivalent performance of the e-learning compared to the lecture based EBM course justifies its implementation from an economic point of view.

While previous research has shown that computer based EBM teaching is equivalent to face-to-face teaching, [12] there has been a lack of studies evaluating educational provision in the workplace via e-learning fitted around doctors' daily practice. A previous study in complementary and allied health had shown that a tailor-made web-based learning experience in EBM was educationally useful, but this was not a randomised evaluation. [13] Contamination of members of the control group in an individual randomised trial tends to shift their outcome in the same direction as the outcome in the intervention group. Thus the effectiveness of the intervention, estimated as the ratio or difference between intervention and control groups, tends to be underestimated biasing trial results towards the null. [8] We used a cluster design that eliminated contamination and employed previously validated and pre-tested tools for outcome measures, thus increasing the validity of our results.

Several issues may raise concern about the interpretation of our findings. Trainees in both groups showed high baseline knowledge for all modules, which left only a small room for improvement in the scores. The pre course scores may have been high due to a pre-existing EBM culture within the participating sites. The modest sample size may have contributed to a limitation in statistical power to detect a small improvement in knowledge between the groups. A small proportion in both groups had shown lower scores post course compared to pre course, but such an observation has also been made in other studies [14] and might be attributed to cognitive test anxiety. [15] Post-course knowledge assessments had been conducted immediately after the lectures in the control group. This may have put the intervention group at a disadvantage as delayed and unsupervised knowledge assessment may have influenced the outcome adversely. Despite these concerns, on average, the intervention group had a tendency towards better performance. We are therefore confident that our findings merit consideration.

Previous studies have shown the value of computer based learning. [12,16-18] Since the costs of delivering e-learning courses after initial investment are small, this may also provide a less costly solution for EBM training than lecture based courses. Moreover, e-learning allows for independent and flexible study, with the possibility of a standardised provision that can be easily updated. The trial's generalisability may be considered limited as participating centres were all obstetrics and gynaecology departments. However, the results of the pilot testing conducted in different medical departments in Switzerland showed that the course was adaptable to different medical specialties. This was further reinforcement of the finding of adaptability across languages and settings in our earlier study. [5]

## Conclusion

We conclude that the clinically integrated e-learning course for basic EBM proved to be at least as effective as a conventional lecture based course and merits consideration for incorporation in on-the-job training for just-in-time learning. Further research should address changes in behaviour after long term follow-up.

## Competing interests

The authors declare that they have no competing interests.

## Authors' contributions

The trial was conceived by KSK, BWM and the Leonardo project team. RK, JZ, BWM, KSK, SFC and JH wrote the manuscript. RK provided central coordination for the project. SFC and JH obtained local regulatory approval. SFC, BWM conducted the trial in the Netherlands and JH, KD, SM, KSK conducted it in the UK. GZ provided internet based data collection for the e-learning group. JZ performed the statistical analysis. All authors were involved in the development of the project and all authors reviewed and commented to the manuscript.

## Pre-publication history

The pre-publication history for this paper can be accessed here:


